# Contemporary chiropractic practice in the UK: a field study of a chiropractor and his patients in a suburban chiropractic clinic

**DOI:** 10.1186/2045-709X-21-25

**Published:** 2013-08-08

**Authors:** Bjorn J Hennius

**Affiliations:** 1 Parkwood Chiropractic Clinic, 93, Cathedral Road, Cardiff CF11 9PG, UK; 2 Welsh Institute of Chiropractic (WIOC), University of South Wales, Treforrest, Wales, UK

## Abstract

**Background:**

Two recent surveys of chiropractors in Great Britain suggest that there are discrepancies between chiropractic practice as defined in regulatory guidelines and day-to-day chiropractic clinical practice and there is in general a paucity of information regarding the characteristics of contemporary chiropractic practice in the United Kingdom. This field study describes the daily practice of a contemporary British UK-trained chiropractor.

**Methods:**

The fieldwork took place during the spring and summer of 2008 when the author spent one day per week observing consultations and interviewing patients in a chiropractic clinic. The chiropractor was subjected to interviews on two occasions. The author also registered as a patient. Field notes were taken by the author, interviews were recorded and the transcripts were corrected and analysed by the author.

**Results:**

A total of 25 patients took part in the study. The interaction that took place between patients and staff in reception could be considered as a prelude to consultation facilitating the transformation from individual to patient and back to individual. Coupled with the continuous physical contact between the chiropractor and each patient there was a substantial amount of verbal and non-verbal communication throughout treatment visits. The patients presented with predominantly musculo-skeletal pain and the majority had consulted the chiropractor as a result of recommendations from others in their close social environment. The majority of the interviewed patients had either an inaccurate or at best rudimentary understanding of the mechanisms of chiropractic treatment. A few of the interviewed patients indicated that they had at first experienced concerns about the nature of chiropractic treatment or getting undressed. The author was able to gain some insight into how the chiropractor's experiences, opinions and beliefs had shaped his approach to chiropractic treatment and how this formed the basis of his clinical modus operandi.

**Conclusion:**

Although no robust conclusions should be drawn from this small scale field study it does show that the clinical chiropractic practice as carried out by this UK trained British chiropractor contains a number of elements described in earlier qualitative studies in the United States, Canada, and Australia.

## Background

Contemporary complementary therapy in the western setting has been the subject of a number of surveys and reviews during the last two decades of the twentieth Century [1-11]. Chiropractic practice in particular has been studied by a number of researchers in the United States [12-14], Canada [15,16], Australia [17], and Europe [18-24]. This research has resulted in a substantial volume of predominantly quantitative data.

Ethnographers have historically taken an interest in the activities of body oriented healers using manipulative techniques in foreign cultures [25-29], but field studies that describe the characteristics of chiropractic clinical practice in western settings are rare. The first ethnography of a chiropractic clinic was carried out in the nineteen seventies in the United States and it portrayed the chiropractor as belonging to a group of “deviant” practitioners [30]. Later studies have suggested that chiropractic purists, or *straight* chiropractors, are in decline [31-34]. The most comprehensive study of chiropractors and chiropractic practice took place in Canada and provided, at that time, a detailed overview of the Canadian chiropractic profession [35].

### Contemporary chiropractic in the United Kingdom

There is a paucity of descriptive and qualitative information regarding the characteristics of contemporary chiropractic practice as carried out by UK trained chiropractors in the United Kingdom. Two recent questionnaire based surveys indicate that there are different approaches within the British chiropractic profession and that the beliefs, theories, and clinical rationale that underpin the practical application of chiropractic treatment vary between individual practitioners [36,37].

The surveys included questions related to treatment efficacy, scope of practice, a chiropractor’s role in healthcare and within society as well as the beliefs underpinning clinical practice. A large proportion of respondents were in the age groups 31–35 years, (16.97%), and 25–35 years, (34%), respectively.

Sixty-eight per cent of respondents agreed with the statement that chiropractors are ‘primary contact practitioners’, and 49 per cent believed that “a chiropractor primarily treats neuro-musculoskeletal and, to some degree, visceral or organic conditions by giving adjustments and using complementary therapy modalities”. Even though 55 per cent of responding practitioners believed that “Mainstream and chiropractic paradigms are compatible in practice” 30 per cent disagreed with the statement “In today’s chiropractic profession science is more important than traditional chiropractic beliefs/philosophy” [37].

The surveys further indicated that 76 per cent of respondents “strongly agreed” or “agreed” with the statement; “Traditional chiropractic beliefs (chiropractic philosophy) are an important and integral part of chiropractic” and 18 per cent “strongly agreed” that “Gastro-intestinal complaints” are treatable by chiropractic methods. 17 per cent of respondents gave the same reply for the chiropractic treatment of “Menstrual disorders/dysmenorrhoea/PMS” [36,37].

This data suggests that there may exist discrepancies between the content of regulatory guidelines on the one hand and actual day-to-day chiropractic clinical practice on the other in the British arena. This field study is an attempt to describe the daily clinical practice of a contemporary British UK-trained chiropractor through the use of participant observation, observations and recorded interviews with patients. Its specific aims are:

•To briefly describe the Chiropractic Clinic and the activities in reception;

•To describe the author’s experiences as a patient during a first consultation and when undergoing chiropractic treatment;

•To describe the characteristics and structure of the treatment visits;

•To explore the reasons why patients decided to consult the chiropractor, patients’ understanding of the mechanisms of treatment, and some of their concerns;

•To explore the rationale and beliefs that form the basis of the chiropractor’s approach to chiropractic treatment and clinical practice.

## Methodology

The study took place in the period between early April and the end of July 2008. The author, who is himself a practising chiropractor, made a conscious decision to approach a chiropractor practicing in another town in order to avoid familiarity with the setting and minimize the risk of meeting former patients. In his mid thirties and with fourteen years in clinical practice *Ken* was a suitable representative of the practicing British UK trained chiropractic population. By the end of the twentieth Century European and British chiropractors were predominantly male and of a mean age in the range 35–40 years [21]. A survey carried out on the British chiropractic population at the start of the twenty-first Century found that 55 per cent of the respondent chiropractors were males in the age range 26–45 years.

Formal consent was obtained after writing an introductory letter to the owner of the clinic, a chiropractor practicing elsewhere. It was agreed that the author could spend one afternoon per week at the Clinic. After a meeting between Ken and the author it was agreed that Wednesday afternoon suited both best. Each session at the Chiropractic Clinic lasted 3–5 hours and consisted of observations of patient treatments and in reception. Semi-structured interviews were carried out with patients in separate rooms between observations of treatments and on two occasions in June 2008 the author carried out interviews/conversations with the chiropractor. The author also consulted Ken as a patient at the Clinic and received chiropractic treatment. In addition to this the author was able to spend time observing patients and the receptionists in the reception area.

Patients attending the Clinic for treatment on Wednesday afternoons were asked by Ken in the treatment room or by the author in reception if they would agree to be observed and/or interviewed by the author. Potential interviewees were taken to a separate room after observation during treatment or straight from treatment and presented with a printed A4 sheet explaining the study, its confidentiality, and its voluntary nature, (see Additional file 1). The author found that a verbal explanation of the study was usually more effective. A maximum of 20 minutes was allocated for each interview and all were based around the following 3 questions, chosen by the author on the basis of a pilot study:

1. “How did you end up consulting [Ken] the chiropractor?”

2. “What is your understanding of what [Ken] does?”

3. “Were or are you concerned or anxious during consultations and treatment? (see Additional file 2)”

Interviews were digitally recorded and later transcribed by a medical secretary in the order that they were completed during the field study. Interviews were not formally coded and all transcriptions were checked and corrected by the author. Patients’ answers to the three interview questions were often embedded in general conversation but were easily located and extracted by the author. Because of the small size of the study and the variation in individual responses to the interview questions the author did not make use of any strict method of analysis. The majority of the replies to the three questions were grouped together for use in table format in the corresponding sections of this document. A small number of responses have been integrated into the narrative of the text or used as quotes. The aim of the author has been to include all answers without exclusion in order to preserve the individuality of the responses and to provide a varied and descriptive account.

During observations in the treatment room and reception the author made field notes using pen and paper. Notes were in addition made immediately following the author’s own consultation with the chiropractor. There were no specific criteria put in place by the author in relation to what to include from the notes and what to leave out when writing this document. The author did however make a conscious effort to select and include material that illustrates the personal and unique nature of the patient-clinician interaction during treatment sessions and at the same time describes the clinical routines put in place by the chiropractor.

Extracts from the field notes made in relation to the author’s experiences on his first consultation with the chiropractor and the “New patient” observed in reception and during a first consultation with Ken were used to strengthen the narrative of the section of this document that describes the “First visit” to the chiropractor. Notes from 3 observed patients were used extensively in the section describing the structure of treatment visits. The 3 patients were chosen by the author as case studies on the basis of patient age, gender and main complaint. Their treatment visits are an integrated narrative of text and extracts from field notes illustrating Ken’s personalized approach to his patients and his established clinical procedures.

Although there is an attempt at integration of information responses during interviews and extracts from field notes were used independently in appropriate sections of this document.

For reasons of confidentiality all patients’ names, as well as those of the chiropractor and Clinic staff, have been changed. The name and exact geographic location of the Clinic have for the same reasons been omitted. Gender and age of patients have been left unaltered.

To carry out a Field study based on participant observation and recorded interviews while adopting an explorative and interpretive approach in the search for the reason for and meaning of observed phenomena would not have been possible without the pioneering efforts under the banner of *functionalism* in early anthropological field work. In broader terms this study may be considered an example of social anthropology [38]. The observation and description of the clinic as an institution and its staff-patient and patient-physician relationships does also contain elements of the *realist* style documentary approach [39].

This study has had ethical approval from the University of Brunel and is based on the data obtained during the author’s research for a final dissertation in relation to an MSc Medical Anthropology.

## Research findings and discussion

There are ethical and practical perils of simultaneously taking on the role both as an observer *and* as a patient in a field study. Carrying out participant observation as a fellow professional has been referred to as “housewife anthropology” [40,41], or even “anthropology at home” [42]. The risk of loss of objectivity and clouding of judgment in this field study is, in the author’s opinion, counterbalanced by familiarity with clinical routines and procedures [43]. The fact that the author was the sole recorder of notes and in addition had editorial control over what material to include and what to leave out is a potential source of bias that must be taken into account in this type of study. The fact that the author had not visited the Clinic before in any capacity did enhance clarity of perception and should on the other hand, in the author’s opinion, be seen as a strength of this study.

### The chiropractic clinic

The Clinic, located on the ground floor of a converted bungalow, was situated in the suburban area of an industrial coastal town in western Great Britain and had been up and running for more than ten years. It was the workplace of 3 chiropractors and an herbalist, and was staffed by 3 part time receptionists working on a rota. The bungalow was situated on the corner of a main through road and had, next to the gate, a large sign visible to passing cars. Trees outside the perimeter provided shade and also functioned as a barrier to the noise of the traffic. A large part of the garden had been covered over with tarmac and another part with gravel in order to provide parking space for patients and staff.

The Clinic entrance, positioned between two bay windows, consisted of a glass door and was usually unlocked. The left bay window, that of the principal treatment room, had its blinds drawn while the right bay window was that of the reception. On entering an inner wooden door led to a dimly lit corridor where the visitor was greeted by the scent of camphor and eucalyptus. There were three treatment rooms on the ground floor and a toilet for patients at the end of the corridor. Carpeted stairs provided access to the converted attic that was now office space and staff area. The Clinic had no x-ray facilities (see Additional file 3 for a floor plan).

### The reception

Immediately to the right from the corridor a wide doorway opened into the reception area, a converted front room. The walls were painted pale beige and five upright chairs were placed in lines, two along the left wall and three with their backs to the bay window. There was a potted plant by the wall to the left and a smaller one on a pedestal next to the window. A low table with magazines stood on the floor and next to it there was a box painted with a picture of Winnie-the-Pooh containing children’s toys.

At the far end of the room the curved reception desk was positioned so that the receptionist was facing the entrance. A PDQ machine was placed on top of an elevated section of the desk and there was a flat computer screen next to the telephone. Another fax/telephone stood next to a tall, grey filing cabinet in the corner behind the receptionist’s back. Next to the plant by the window there was a water cooler and a radio/CD player on a stand providing a background of the small talk, news and music characteristic of BBC Radio 2.

On the shelves above the reception desk there was a display of neck pillows and lumbar supports for sale to patients. A row of information brochures stood upright in a plastic holder fixed to the wall next to the reception desk. A framed poster, depicting a floating iceberg representing symptoms as its visible ‘tip’ and the hidden causes below the surface, hung on the wall above the brochures. Smaller framed pictures of landscapes were mounted on the wall separating the waiting room from the corridor. Two framed price lists were mounted on the wall behind the receptionist informing patients that the price of the first consultation was £45 and that subsequent treatment cost £27 per session.

In reception patient details were registered on the computer by the receptionist but patient records were handwritten, as was the Clinic diary. When patients phoned in order to book appointments the receptionists would provide information on when the three chiropractors were on site, times available for appointments, and the cost of a consultation.

The clinic management had decided to actively start selling products to patients on the condition that the item was recommended by the patient’s chiropractor. Sandra, one of the receptionists, was constantly re-arranging and improving the presentation of ice packs, neck pillows, lumbar supports, and other items on display.

In addition to its function as the administrative and financial hub of the Clinic the reception was a place for social interaction. Sandra chatted happily with patients and her volleys of laughter could be heard in all parts of the building. Megan, the other part time receptionist that would sometimes cover on Wednesday afternoons, was less vociferous in her approach but with her warm smile and discrete sense of humour she was equally good at making patients feel at ease. Patients in pain were always given sympathy and support, both from staff and other patients. Although not specifically a question set up as part of the interview protocol the nature of the reception area gave rise to a number of comments from patients (see Table 1).

**Table 1:** Patients’ comments about the reception

**Name andAge**	**Patient’s comment with regard to the reception:**
**Vera, 46**	*“I didn’t know what to expect or as you say I hadn’t been to a chiropractor*. *It’s a very nice environment*, *very calm and friendly…*.*I only know [Ken]*, *[chiropractor]*, *I never had any treatment through anyone else…*.*The receptionists are friendly*, *good…Good environment*.*”*
**Ron, 30**	*“It’s fairly relaxed*. *It’s not a clinical atmosphere…*.*You’d probably liken it to a dental practice rather* (*than a*) *hospital environment*. *So*, *it*, *yeah*, *it’s fairly relaxed*.*”*
**Pat, 26**	*“It looks something like a surgery because you have a reception that you need to sign into and then chairs there……and I had to sign some papers and had some general chit chat with the receptionist…*. *I felt quite relaxed…*.*And quite warm colours and…*.*the sun was shining and lots of light coming in and…there’s magazines to read*. *It just relaxes you before*.*”*

Staff and practitioners were all on first name terms and the receptionists referred to the chiropractors by their first names when talking to patients. New patients were formally addressed as *Mr* or *Mrs/Miss* followed by their surname but after a few treatments formal tone was usually dropped. If the chiropractor was running late tea or coffee was often offered to waiting patients. Conversations in the reception were usually initiated by the receptionist and topics ranged from aches and pains to music, travel, and the youth of today. Specific details of treatment were generally not discussed although patients did at times comment on the firmness of a chiropractor’s manual techniques or how they were feeling afterwards. Most seemed pleased with their progress.

As the first point of call for anybody entering the Clinic, the reception induced a feeling of welcome and calm. It has been suggested that the layout of the reception, as well as its furniture, scent, posters, pamphlets, sounds and products for sale all have a strong symbolic function. Arriving, registering, and sitting down to wait has been described as part of a ritual with important social and psychological dimensions for the individual as an environment facilitating the transition from *ill person*, (person in pain), to *chiropractic patient*[44].

#### The chiropractor

Ken was a 1994 graduate of the Anglo European College of Chiropractic, (AECC), Bournemouth. As a member of the cohort that graduated in the year that the *Chiropractic Act* went through Parliament he had at first started out in another established clinic where he was able to make the transfer into clinical practice under the guidance of an experienced senior colleague. A survey in the early part of the twenty-first Century indicated that close to fifty per cent, (49.3%), of chiropractors in the UK worked alongside other chiropractors, and a third worked with other alternative or complementary practitioners. The survey further suggests that the vast majority of British chiropractors treat their patients in a clinic and only 5 per cent see their patients exclusively at home [36]. After moving on Ken had successfully built up a reputation of being a competent chiropractor who was liked by his patients. Despite the long hours, the routine of clinical procedures, and the responsibilities as a health care professional I was given the impression that Ken liked his job. I was aware that my presence in the treatment room as an observer might interfere with the patient-clinician interaction and assured Ken that I would do my utmost not to disturb. Ken said that he was happy to have me in the room as an observer provided that the patient had given consent.

Apart from my account of my consultation and treatment a total of 13 patients, 8 females and 5 males with ages ranging between 27 and 89, agreed to be observed.

#### The first visit to the chiropractor

I parked my car on the road outside the clinic just after 2 p.m. on a sunny Wednesday afternoon. The reception was quiet and there was only one patient, a woman in her fifties, waiting for her appointment. We were both offered a cup of tea by Sandra the receptionist and we both accepted. Sandra was busy entering data on the computer and answering the phone but had time to chat, generally about the sunny, but chilly, weather. I asked about payment and was told that it was normally taken after the consultation. Despite the music on the radio I could hear the muffled sounds of conversation in the treatment room across the corridor behind me.

A younger colleague of Ken’s, a man in his late twenties, came into reception in search of a patient file. He wore a white, short-sleeved clinic jacket with the Clinic logo printed in blue on the breast pocket. Ken was running late.

When finished with the previous treatment, Ken, wearing a white clinic jacket, came into reception and shook my hand. He was given my patient records by Sandra and led me into the principal treatment room across the corridor. I was told I could bring the cup of tea with me.

### The treatment room

On entering the treatment room the domiciliary nature of reception was immediately replaced by a distinct clinical atmosphere; Closed blinds ensured privacy, the light was subdued, and the scent of medicinal herbs was stronger. On a narrow office table along the far wall there was a switched off x-ray viewing box, a computer screen, and a keyboard. There were academic books on the shelves and framed diplomas hung on one of the walls. Charts showed anatomical details of the human skeleton, muscles, and the nervous system. A partly drawn circular curtain near the window concealed a stool and some folded patient gowns. Instruments for examination including a reflex hammer, a stethoscope, and a sphygmomanometer, were laid out on a small table. From a metal stand on the floor hung a life-size bone coloured plastic spine. The air was comfortably cool and there was no background music.

The space in the centre of the room was taken up by an imposing structure in leather and steel: the treatment table. Padded dark blue cushioned sections only partly concealed the complex metal machinery underneath. Electrically operated pedals, switches, and a set of levers controlled the mobility of the table sections. Thin white protective paper on a roll by the headpiece provided comfort and hygiene for patients.

Ken closed the door and I was invited to sit on the chair by the table. There was a feeling in the room of privacy. It was an environment distinctly suited to investigation, examination procedures, and treatment. Ken took up his position in his office chair and looked at my file. Sandra had assumed that I had filled out my personal details at our previous meeting. Ken was unperturbed and asked me to start filling in my personal details and medical history. While Ken left to make himself a cup of tea I proceeded with the task of revisiting my life in the light of pains, occupation, habits and lifestyle as well as past ailments, accidents, operations, and treatment.

### Taking the case history

Ken read through what I had written on the form and asked questions. I explained that my general health was good. After informing me that he primarily worked as a musculoskeletal specialist and that any other health concerns affected would be a bonus not planned for, he asked me what I had come to seek help for. I explained that I had a long-standing, reoccurring left-sided neck and shoulder pain. I pointed to the area and Ken took notes. After asking about the time of onset, progress, pain patterns and any other symptoms he explained that he needed to carry out an examination. Ken seemed relaxed throughout the procedure and I had the impression that he cared about what I was saying and that he was interested in what I was telling him. Interest and compassion are both factors that have been demonstrated to have an effect on patient satisfaction [45-47].

The intelligent, sensitive, and systematic collecting of information from patients has been identified as a fundamental characteristic of the clinical encounter [48]. Arguably constituting the most important element of the first consultation, the clinical interview/case history taking also has therapeutic functions on a number of different levels. It has been suggested that taking the case history represents;

1. Communication about the condition [49];

2. A demonstration of caring and developing trust [50];

3. A narrative of the patient’s experience of the condition [51,52]; and,

4. A start in the formation of a genuine relationship between the clinician and the patient [53].

Apart from the clinical importance of exploring the “archaeology of a person’s past”, it has also been suggested that the case history taking allows the patient to experience past memories [54]. The patient interview has even been described as a performance not unlike improvisational jazz; The experienced clinician asks questions, reacts to, and follows the patient’s responses in a negotiation of *medical meaning* together [55].

### Examination and treatment

Ken suggested that he would concentrate the examination on the shoulder/neck area but include a screening of my low back and lower limbs. I was asked to empty my pockets and remove all clothing apart from trousers and underpants. While undressing I fleetingly felt self conscious about the fact that although I had showered and was wearing clean clothes the car journey in the heat had made me perspire. My notes from the examination are full of detail:

*“Examination started with me standing, facing the window. The room was cool but not cold and I was conscious of my own posture and leg stance. I was touched around the neck shoulder area, palpated, and asked to turn and tilt my head. Ken asked whether any of the procedures caused pain and I told him when I felt pain. Ken checked my toe flexibility and leg*/*ankle*/*knee alignment and I was asked to place my hands on the door and do a high stepping gait while he palpated my lower back*. *My finger floor distance was measured*.

*Sitting tests consisted of neck range of motion*, *nerve stretch tests*, *compression and distraction*. *Neurological tests* (*reflexes*) *where done and I was continuously asked regarding symptoms*. *At certain procedures I felt very sore and achy in areas under my skull*, *by my left shoulder blade and on deep pressure of the neck vertebrae*.

*The reflexes* (*upper and lower limbs*) *hurt as the rubber hammer hit Ken’s thumb and my tendons*.*…*

*Finally I was asked to lie on my back and my neck and head alignment were examined*. (*Deep pressure on my nerve roots from an anterior direction*). *I was told that my head was not sitting ‘straight’* (*Ken asked “I don’t know if anyone has said this to you before but…”*).

*Ken asked me to sit up again facing the desk and the x-ray box*. *He proceeded to outline his conclusion:*

*“There are areas of your neck and upper thoracic spine that need treatment*. *Although neck range of motion is generally good there is a low* (*Cervico thoracic*) *‘dysfunction’*.*”*

Ken explained that he proposed to treat me using manipulation of the joints and soft tissue work. He asked me if that sounded ‘ok’ and I said I was fine with that. This stage in the consultation has been referred to as the “Explanation to the Patient” or “The Plan of Treatment” [35]. Ken then told me to lie on my back on the treatment table. The following extract from my notes describes the first treatment:

*“Ken proceeded to traction my neck*. *The stretching action was carried out with his hands firmly gripping my chin and back of the skull*. *The stretch was comfortable*. *After this Ken proceeded to do sideways rotational stretches using* (*his*) *arms elbows and hands*. *I told him that the stretch felt firm but that it was comfortable*. *A more segmental rotational stretch*/*mobilization was carried out after deep finger pressure on the left side of the upper vertebrae at the base of the skull…*

.....*Painful – up into my head*, *but abating*. *Ken explained that he was going to ‘click my neck’ and asked if I was OK with that*. *I said ‘Yes’ and he manipulated my upper left neck*. *There was a slow turn to the right*, *then a quick turn*, *and another*. *After one or two turns there was a loud click behind and under my left ear*. *The point of contact in the left upper neck was sore and I had expected more pain*.*”*

Ken treated my neck on the other side and then asked me to turn over onto my stomach.

*“He walked to the door and put a lubricant on his hands and then proceeded to massage*, *deeply*, *my shoulder and neck region*, *concentrating on the left side*. *The treatments were at first painful*/*achy but felt better as he continued the procedure*.

*Ken explained that I had a misalignment in the cervico thoracic junction and that he had chosen to treat the region posterior to anterior*. *He again asked me if I was alright with having the treatment and I said I was*.

*Ken asked me to inhale – then exhale*. *As I let the air out he applied slow pressure with his hands placed on the upper part of my back*, *at the thoracic spine below the base of my neck*. *Suddenly he pushed down*, *fast*, *and there was a moderately loud ‘crunching’ sound but*, *to my surprise*, *no pain*.*”*

During one of our conversations at a later date I remarked on the fact that Ken seemed to use a lot of massage prior to manipulation treatment. I recorded his answer:

*“Yes*, *I perhaps use…*.*I use it more judiciously now and that would*, *I would try and ask myself what I can do best for this patient? What will be the best thing? What will give the greatest therapeutic gain to this patient? And I think soft tissue work is part of that…*.*soft tissue or muscle mass represents a greater amount of mass than mere nerve endings in facet joints…*.*And they are the work horse of the body and you need them on your side…There is going to be a protection of that area and you’ve got to persuade the body that it’s…*.*what you intend to do is ok*.*”*

As treatment was completed Ken went on to assess the effect of treatment, a process sometimes termed *evaluation*[35]. My neck and shoulder area felt relaxed and warm. My notes describe the final stages of my first visit:

*“My face felt hot and compressed but there was a feeling of warmth and comfort in my neck*/*shoulder and head*. *Ken proceeded to test my neck rotation and I could feel a noticeable difference in flexibility and comfort*.*”*

The first consultation had taken just over an hour. I remember that Ken’s hands had felt warm, firm, and reassuring throughout the first visit. A recent survey showed that chiropractors in Great Britain spend 31–60 minutes with a patient on the first consultation, and 15–30 minutes on subsequent visits. 68 per cent of the responding chiropractors indicated that they always required the patient to undress down to their underwear on the first consultation, and 42 per cent replied that this was the case on subsequent treatment visits [36].

I was asked to dress and followed Ken into reception where I arranged my next appointment through Sandra and made a card payment of £45.

### Observation of a patient visiting the chiropractor for the first time

#### Eric, 89, a ‘New’ patient

Eric sat in a chair in reception with his back to the window. I took notes:

*“The ‘new’ patient was talking to himself and his grandson about his blood pressure and the medication taken for it* (*high b*.*p*.). *he continued to talk*/*mumble and his grandson helped clarify the questions asked on the form*. *The young man was embarrassed* (*appeared to be*) *about his older relative’s behaviour*, (*talking*/*mumbling aloud*, *fussing about what to write*, *etc*.*…*)

*…*..*The elderly man continued in his gravelly voice*. *He referred to his medication*, (*“The GP wants to preserve me a little longer”*).

*Ken came out to fetch the new patient who excused himself for not ‘leaping’ up when standing up to shake hands*.

*Ken said: “That’s alright*, *it may not be advisable”*.

*They walked together into one of the treatment rooms*, (*the one at the back*). *The new patient brought his stick into the room*.*”*

Eric gave his permission for me to observe his consultation with Ken and I proceeded to the treatment room. The elderly man, looking remarkably fit and healthy for his age, explained that he had a severe pain in the left upper back and was unable to stand up straight. He had hurt himself gardening and then gone from bad to worse by playing golf:

*“Ken was sitting hunched forward*, (*mimicking the gentleman*), *facing Eric*.

*Ken was asking questions*. *Eric was describing a knee injury*, *that he had been on Meloxicam*, *then*, *told not to be on them for long*, *changed to Co-codamol*.

*Ken started to ask detailed questions*, (*“Does it hurt when you cough?”*, *“Have you had this before?”…*.) *Ken was speaking loudly*. *He went through previous diseases*, *asking details*. *The patient commented*, *on his spelling*, *that he left school 75 years ago*.

*There was lots of humour*, *laughter*, *irony*. *Ken asked: “Anything else I should know?”*.

*Eric spoke of chest x-rays – “a shadow”*. *“I never smoked in my life”*, *“it must be the hay”*.

*Ken commented: “We need to pin this down”*.

*Eric said he wanted to know what was wrong*. (*He*) *described how he had held on to the lawn mower*. *Eric said “Golf probably made it worse*. *Couldn’t let the young ones get away with it”*.

*“Only 4 of us left*.*”* (*Comment on his age*)

*“Still bloody minded by the looks of it” said Ken*.

*“Competitive” replied Eric*.*”*

Studies have demonstrated that when clinicians use empathic and patient centred communication styles in a stress free environment this may have a positive effect on treatment outcome [56]. When Ken was satisfied that he had enough information he asked Eric to remove his top in order to prepare for the examination.

*“Ken demonstrated Eric’s stoop*/*antalgia* (*by placing him*) *in front of the full length mirror*. *Ken then asked Eric to take a seat* (*on the treatment table*), *and asked him to specify the left area of pain near the low back*.

*Ken started palpating standing behind Eric on the left*. *Ken located a spinous process in the thoraco lumbar junction that was tender*.

*Ken proceeded to percuss the spinous processes*, *using a reflex hammer*. *Eric said: “You’re playing on the xylophone*.*” Ken laughed*. *Ken then brought a tuning fork from next door*.

*“I’ve got a cold piece of metal here*.*” Ken hit his knee and placed the tuning fork ‘foot’ on the spinouses*.*”*

When Eric was examined Ken took care to use a more simplified vocabulary. Ken carried out some further tests on Eric while simultaneously explaining in lay terms what the tests were for. Seeing the difficulty that Eric had in moving around Ken then arranged to swap rooms with his younger colleague in order to be able to use the *hi-lo* function on the treatment table in the principal treatment room. This mechanism allows the treatment table to rise into a near vertical position enabling elderly patients and those in severe pain to step onto a footplate and be gently lowered into the horizontal position.

#### Eric’s treatment

I followed Ken and Eric into the principal treatment room where the elderly patient was assisted onto the footplate at the base of the vertically elevated treatment table. Ken pressed the foot pedals. There was a humming noise as the treatment table slowly lowered to a nearly horizontal position. Ken explained his findings and the proposed treatment at the same time as he was helping Eric into a comfortable position. Eric was lying on his front:

*“Ken explained that he had concluded that* (*Eric*) *had strained a muscle in the left lower back*.

*Eric asked: “Will it eventually heal?”* (*there was a discussion of the body’s ability to repair itself and the effect of age*)

*Ken explained that he was going to start working on the muscle and then* (*that he*) *would stretch it*. *Ken kept asking “Ok?” to get the patient to make a choice*/*agree…*..

*…*..*Ken continued to work on the left muscles of the back and then raised the table up to vertical*. *Eric was asked to turn to his side* (*right side against the table*). *The table was lowered to horizontal and Eric was stretched in ‘side posture’*. *He [the patient] indicated that there was some pain as Ken applied pressure*. *Ken was at all times close and making sure that Eric was stable*.*”*

At this point Ken stood back and explained that this was as much as he would do on the day. It was agreed that Eric should arrange to return in 2 days. Ken instructed the patient not to play golf the following day. Eric had some questions:

*“Eric asked about time and how many treatments would be needed*. *Ken explained that he would expect full recovery*, *but that it would take some time*, *albeit without numerous treatments*. *Eric’s concerns were voiced*, *and Ken*, *although conscious of time* (*the next patient was waiting and he was delayed*) *made efforts to explain and answer questions*.*”*

When back in reception Eric paid and made an appointment for Friday morning.

### The chiropractic consultation

It has been suggested that the chiropractic consultation consists of four separate stages:

1. Acceptance/Validation

2. Expectation/Explanation

3. Clinical Action

4. Engaging Plan [57]

Coulehan goes on to say that stage one revolves around positive regard, empathy, (“the ability to sense the patient’s experience and feelings accurately, and to communicate that understanding to the patient”), and genuineness. He also states that “fulfilling expectations by using concrete, understandable explanations is the second movement in the chiropractic process” [31]. Helman [44] suggests a not dissimilar staging of the initial healthcare consultation:

1. The patient’s presentation of *illness*.

2. The interpretation and translation, by the practitioner, of the presented illness into *disease*.

3. The prescription of an appropriate *plan of management*, (this could be treatment or referral), that is acceptable to both the practitioner and the patient.

Stages 2 and 3 are considered to be of particular interest to the patient and this is where the communication between the practitioner and the individual seeking help is crucial. Studies have shown that patients in general have a number of set questions that they want the answer to:

1. What is the cause of my pain?

2. Can it be treated (by you/a chiropractor)?

3. What will the treatment consist of?

4. How many treatments will I need?

5. How long will it take before I get better [44,58,59]?

In addition there may be questions such as; What will happen if I don’t have treatment?; Why has this happened to me?; What will be the cost of treatment?; and, Will this happen to me again after I have finished treatment? This set of questions has been referred to as the *patient’s agenda* and the *patient explanatory model*[44,60].

Kaptchuk has pointed out that the initial chiropractic consultation almost invariably results in a firm diagnosis that “matches patient perceptions” [61]. Kelner found that 84.9 percent of the chiropractors always explained the nature of the illness and 88.9 percent explained the treatment to patients [35]. A Finnish study has suggested that being told a diagnosis can in itself be a source of great relief to patients suffering from back pain [62].

Coulehan concludes that “Chiropractors ‘do something’.”. This includes the extensive spinal examination involving “much laying-on-of-hands” in the third stage, the *Clinical Action*[31]. Touch is generally considered a natural element in the caring for others and some authors believe that it is directly related to enjoyment and health [63,64]. It has been suggested that the prevalence of touch in the clinical encounter gives chiropractic an advantage over other forms of care where there is little or no physical contact between the clinician and the patient [54]. Kaptchuk suggests that one therapeutic advantage of chiropractic is that there is invariably an *intervention*[61]. Coulehan is of the opinion that manipulations are dramatic events:

“…..Successful completion is indicated by a snap, click or pop audible to the patient [31].”

One of Ken’s patients, Owen (aged 36), illustrated these points when I asked him about the difference between seeing his doctor and seeing the chiropractor. He replied:

*“GPs seem to be almost reluctant to do any hands on care*. *They prescribe from a distance sort of thing whereas when you come here they will check you out and they will do things*.*”*

#### The structure of the treatment visits

In the treatment room Ken was invariably on first name terms with his patients. Treatment visits seemed to follow a pattern starting with the greeting in reception followed by going to the treatment room. The consultation started with questions, then continued with undressing, examination, treatment, re-assessment, and advice. Treatment sessions consisted of a lot of hands on activity and were highly tactile. Ken’s hands would deliver a mixture of discomfort and pain relief, always with therapeutic intent. I noted that Ken, as part of the examination, carried out *static* and *motion palpation* on all patients prior to starting treatment:

*“Bjorn: “So are you checking for mobility*, *muscle tone* (*or*) *are you looking for alignment*, *or all of those?”*

*Ken: “Yeah*, *I am looking for smoothness of movement*, *range of movement*, *resistance to motion palpation if there’s a muscular resistance or perhaps* (*an*) *articular or ligamentous resistance*. *That would make me feel like more like manipulating that area If there’s a firm* (*feel*)*…*..*If there’s a sort of spongy muscular resistance*, *I don’t think they respond quite so well to manipulation and more to massage and stretching*, *trigger points or manipulating those* (*joints*) *where that muscle might cross*.*”*

Ken pointed out that I would become aware of patterns in his routines, such as the fact that he nearly always started treatment with traction. At one point Ken suggested that chiropractic treatment not only involved manual procedures but an equally important element of verbal communication:

*“Ken: It’s hands on therapy*, *but you’ve still got to persuade each and every patient to get better and they do need encouragement*. *That’s part of what the interview process is about*, *that’s part of every recapping when I meet somebody…’M’*, (*a patient seen earlier*), *for example*, *it took me a few minutes of gauging how she was…*..*…A chat at the beginning to determine how she is feeling*, *what she’s been doing about it*, *what her expectations are…*..*There is more communication than* (*barely the*) *physical action of doing it…So*, *you have to persuade people to get*, *and I think that might be the part of what you learn in the time*. *Not just that you have seen lots of cases of a certain complaint and you see the varying ways that that can respond…*..*Part of the experience in practice is learning how to persuade people to get better…*..

*…It’s learning what people need from you*, *what interaction they need from you…physically and communicatively in conversation and the rapport…the shared experience*.*””*

In my notes I comment on the fact that the complex interactions that take place during a consultation and treatment session are only partially visible in the patient records:

*“In the patient notes I could tell that only the very skeletal aspects of treatment* (*subjective comments*, *segments treated*, *advice*) *were written*. *The consultation contained so much more than* (*just*) *what the S*.*O*.*A*.*P*. *notes would show*.*”*

During my sessions inside the treatment room I witnessed numerous verbal and non-verbal exchanges and interactions that were not and could not realistically be recorded by the clinician in the patient records. Jamison found that patient records consisted “largely of notations of biomechanical assessments and interventions” [33].

### Observations in the treatment room

On busy days Ken would alternate between two treatment rooms so that his time could be effectively spent on treatment. The receptionist would show each patient into the vacated treatment room and then leave to let them get undressed. As I returned to the reception after observing a patient I would be told whether or not Ken’s next patient had given consent for me to be in the room as an observer. The treatment sessions consisted of a series of events and interactions specifically tailored to the individual patient but always set against the framework of Ken’s clinical routines. The following are three examples of patients of different ages and gender.

### Brian, 32

Brian was self employed and worked with computers. He was suffering from low back pain and had been treated by Ken the previous day. When I entered the treatment room Brian was naked from the waist upwards. Ken carried out a number of orthopaedic tests checking for range of motion and pain.

*“Ken:*,(*…as patient stands with hands on door*, *high step marching on the spot*) *“It’s moving nicely*, *fella!”* (*Commenting on low back*/*pelvic movement*)

Ken often had what may be described as conversations with the tissues that he was examining or treating. Sometimes it would seem as if the patient functioned as an interpreter and answered back.

Brian sat on the treatment table and Ken, standing behind him, palpated the low back firmly in a forward and backward motion making the patient’s abdomen move with each push. There followed some orthopaedic tests with Brian lying on his back and then on his front. Ken’s hands rarely left the patient’ body as he talked his way through the examination, asking questions, touching the relevant parts of the body, and waiting for answers. Oths noted in her field study that there was a substantial exchange of information during examination and treatment procedures [34].

Ken went to the shelf, put massage oil on his hands, made sure his hands were well oiled up, and proceeded to massage Brian’s lower back. In contrast to the first part of the visit there followed a full minute of silence before Ken spoke:

*“Ken starts discussing walking [the Dog]*. *Ken asks what dogs Brian has*.

(*The patient replies:*) (*one Labrador – one Collie*).

*“Crikey!”* (*Ken’s comment*)*”*

As Ken re-examined the low back and thoracic region he again ‘talked’ to the tissues:

“Ken says “Just going to ask the question of this ‘glut’ here”…

*…*.. *“How are you doing?” As he [Ken] presses firmly in*.

*Brian answers* (*“Not too bad*.*”*)

After carrying out the re-assessment Ken made a positive comment:

*“It looks heaps better than when you first came in*.*”*

After wiping excess oil off Brian’s back with a tissue Ken then asked him to turn onto his right side in preparation for treatment. With the patient in this position Ken flexed Brian’s left knee, placed his left hand on the patient’s left shoulder, and placed his right hand on the low back. Brian was asked to breathe in and then out. Ken then carried out a *side posture* manipulation rotating the low back region and thrusting with his right hand on the lumbar spine. The procedure was repeated with Brian lying on his left side.

*“With Brian supine Ken goes on to explain the healing process*, (*“the patient does the healing”*), *and explains ‘his’ job*, (*as a chiropractor*).

*Ken suggests that Brian should give the complaint approximately a week*. *Then decide* (*if the condition worsens*) *to come back*.

*Ken emphasized the ‘nipping’ in the bud*. *Brian asks “Your advice on how often to ‘pop’ in?”*

*Ken replies: “Young healthy bloke like yourself? Quarterly…”* (*Seasonally*)

*Ken concluded by giving advice to keep away from heavier activities*.*”*

In the reception Brian and Sandra discussed the date of his next appointment. Brian was pleased as he had responded rapidly. Oths writes:

“…treatment for long-term poorly defined problems is liable to be a discouraging and frustrating experience……The chiropractor anticipates these difficulties by providing the patient (with) a structured, supportive environment and theoretical explanations designed to take the mystery out of process and problems. This provides the patient with a greater sense of personal control. The resultant effect of the communication characterized by a high degree of information and positive affect is a strong doctor-patient relationship and a satisfied patient who continues treatment [34].”

Oths further suggests that through realigning the framework of the patient’s belief structure to that of *chiropractic* the chiropractor provides an “easily understandable and culturally compatible explanation for heretofore vaguely defined health problems” [34]. Through this process the chiropractor and the patient reach a common level of understanding that should result in patient satisfaction.

### Clare, 27

Clare worked in finance and was a keen horse rider in her spare time. She had been undergoing treatment with Ken for over two months. Her headaches had worsened again during the morning:

*“We enter as Clare sits*, *in her blue gown* (*on the treatment table*)*…*

*Ken checks the neck range of motion and notices a ‘bite’ in the upper thoracic area*. *Clare is asked to lay supine*, *knees bent*, *and Ken starts tractioning the neck manually*.*”*

Ken sat at the head of the treatment table and applied firm finger pressure under the right side of Clare’s head for several minutes. Clare, visibly in discomfort, asked questions about the muscles. Ken pointed out that although they were tiny they were the source of considerable pain. He then rocked Clare’s head gently, using his left hand, turning it in right rotation. He kept the fingers of his right hand pressed firmly into her right upper neck:

*“Moving the right hand down to the shoulder* (*right*) *Ken pushes into another muscle*.

*Clare says it’s ‘painful’…*.. *describes that the pain is going to her head*.

Ken: “Is it the pain that you are getting?”

Clare: “Yes…”

Ken: “Good…”

Ken retracted his statement, (*“It’s not good*, *but…”*), explaining that he was pleased not because he was able to reproduce the symptoms of the complaint but for the fact that he had found the root of the problem. It has been suggested that the pain caused by certain treatment procedures is often experienced as positive and as a necessary part of the therapeutic process by patients [44]. During treatment Ken asked Clare about her work routines:

“Ken: “So what’s the longest you’ll stay at your desk?”

Clare: 3–4 hours…”

*Ken: “What!?” [He] shows surprise at the length of time described by Clare*.*”*

Ken moved on to diagonal stretches, flexing the cervical spine with Clare’s head resting on his forearms. Both sides were stretched. My notes describe the spinal manipulation:

*“Ken moves [the] head from side to side* (*facing left*) *and says: “I’m just going to loosen this one…” He pulls*/*twists the head to the left*, *there’s an audible ‘click’*, *and Clare says: “I could feel that one…” A similar treatment is done to the right*, *and Clare is then sat up*.*”*

Ken went on to treat the thoracic region, with the patient lying on her back on a padded board. Clare was then asked to turn over. Ken applied massage oil to his hands:

*“Before starting he unhooks the bra strap and spreads the gown to the sides*. (*Ken explains what he is doing*).

*Some joint clicking*, *during the massage*, *make Clare and Ken giggle*.

*Ken gets a piece of tissue and does ‘rotational’ manipulations along the thoracics*.

*Ken explains that Clare needs to ‘shimmy’ up 2 inches*, *holds Clare’s shoulders and moves [her] upwards*.*”*

Clare sat up and was asked to do a shoulder stretch, mimicking Ken’s demonstration. He finished by suggesting that Clare ought to do some neck stretches. We left the room and let her get dressed.

### Dora, 51

Dora worked on the till in a local supermarket and kept physically active with yoga, long walks, and walking holidays. She suffered with low back pain and had first consulted Ken four months previously. Dora was receiving supportive treatment every six or seven weeks.

*“Ken asked her to stand against the door*, *doing a high stepping gait*. *In the procedure*, *Ken examined the low back*. *Seated*, *Ken examined cervical rotation*, *and the patient was asked to lie supine*. *Dora talked about doing walks in Whitby*, *Yorkshire*. *She described the mist*, *the cold on the Yorkshire moors*. *Daily walks*, *10–13 miles per day……As Ken was attempting to adjust the cervicals supine he made Dora relax by asking her to ‘twiddle her fingers’*, *‘turn her ear to his hand’*, *and ‘move the shoulder blades together’*, *to ‘occupy herself’ and make her relax her neck*.*”*

Dora was then asked to turn onto her front. Ken massaged the low back and shoulder area. There was talk about travel. I understood that Dora had met Ken’s daughter. The patient went on to describe her yoga schedules as he treated her using *petrissage*, a gentle pummelling massage technique, in the region of the upper back.

Continuing his treatment of Dora Ken aimed his massage at the deep muscles of the left side of her lower back. Dora was concerned that her activities were the cause of her aches and pains. Ken replied:

*“Ken said that the only way that muscles were kept strong and healthy was through activity*, *enforcing the positive benefit of training*/*activity*.*”*

Ken went on to treat *trigger points*, (tender points/areas of tight muscle), in the buttock region, using finger pressure. He concluded the treatment element of the visit by carrying out long lever *helicoidal* stretches with the patient lying in side posture. Using the heel of his hand he applied firm pressure along the length of the lower back muscles on each side of the spine. Dora was asked to sit up and commented that she “would probably be stiff after treatment”:

*“Ken finished with a seated check-up of the neck and upper back*.

Dora asked: “Any do’s and don’ts?”

*Ken said: “Only do’s*.*”*.*”*

I thanked Dora for letting me observe her during treatment before Ken and I left the room. On entering reception I noticed for the first time a sign informing patients that the treatment fee would be increased to £28 on the 2nd of June.

### The patient interviews

Of the seventeen patients that agreed to be interviewed 12 were female and 5 male. The female patients were in the age range 26 to 60 with a mean age of 42. The males were in the age range 28 to 46 with a mean age of 36.4. Despite the modest size of the cohort age ranges reflect the data obtained in a recent survey which indicated that the majority of chiropractic patients were in the age range of 21 to 60, [36]. Interviews would last between 10 and 20 minutes. Some patients provided brief and concise answers while others gave lengthy responses to the interview questions. Only 4 patients indicated that they had concerns in relation to the treatment visits [Question 3: ‘Were or are you concerned or anxious during consultations and treatment?”].

### Factors that make patients seek chiropractic treatment

The processes underlying why and how people seek help are complex. There are a number of factors that influence whether individuals seek professional or complementary care, or at all. It has been suggested that although choice plays a part decisions are made by the individual interacting in social networks that provide mechanisms for learning about, understanding, and handling difficulties [58,65]. Pescosolido discusses the role of contact with others:

“[individuals] continue to ask advice and seek help from a wide variety of lay, professional, and semi-professional others until the situation is resolved or options are exhausted….Illness triggers a dynamic, social process of coping. It is through contact with others that individuals deal with situations of medical uncertainty and find ways to solve emotional and physical problems [66].”

Chiropractors are private practitioners and there is no set referral protocol linking their services to the conventional health sector. All of the patients observed and interviewed during this field study had suffered from pain or discomfort that was musculo-skeletal in origin and which had started to affect their life and daily activities. Ken’s own opinion was that *pain* was what brought the majority of his patients through the door in the first place. Patients were asked about how they came to seek help at this particular Clinic (see Table 2).

**Table 2:** The reasons behind the patients’ seeking a consultation with Ken the chiropractor

**Name andAge**	**Reply to the Question: “How did you end up consulting Ken the chiropractor?”**
**Tanya, 54**	*“I just knew that General Practitioners are not very good with bones and joints and muscles so…I can’t remember how I found out about Ken…*.*I think somebody recommended him but I can’t remember exactly how now*.*”*
**Uma, 60**	*“I’ve been to different things…I have tried one or two different things and I was talking to a neighbour and* (*she*) *recommended me to this clinic*.*”*
**Clare, 27**	(Clare had suffered from headaches and seen her G.P. who had recommended her to see a chiropractor)
	*“The doctor said…The pain in my head was coming from my neck and a friend referred me*.*”*
**Vera, 46**	(Vera was recommended by a colleague at work who was himself a patient of Ken’s) *“Well*, *probably you noticed that I knew the gentleman in the waiting room…Ken is quite well known in our office and it was just a recommendation by somebody to come here*. *And it has sort of spread around the office*. *Most people* (*who*) *have got problems or need to see a chiropractor come here*.*”*
**Nadine, 28**	*“****Bjorn:****…*.*Did someone recommend you…?*
	***Nadine:****No*, *just in the phone book*, *the yellow pages…*.*And just sort of rang up here…*
	***B:****OK*. *Have you been to a chiropractor before?*
	***N:****No*.
	***B:****So how did you know you had to go to a chiropractor…?*
	***N:****Because my mother goes to a chiropractor…*.*”*
**Pat, 26**	*“I just spoke to my partner…*.*He was fed up of seeing me clicked all the time so he said go and see someone about it*, *so I just did a search on the web*. Chiropractors in *******. *This clinic came up and then I asked around if anybody came here or if they could recommend…*.And a friend of mine had gone to a clinic in ***** and said that he was really good, *that she* (*had*) *only one treatment and then she was really happy*, *so I booked an appointment*.*”*
**Sid, 46**	*“It came through my aunt*. *She goes horse riding and she gets problems with her back and she said…Well*, I use a chiropractor in *****, *near where she used to live*. *So*, *I thought it’s as good as any*, *I’ll go there*.*”*
**Irene, 55**	*“My husband has a slipped disc*.…So we were recommended by a friend to see Ken when he worked in *********. *When he did so well with my husband*, *because he sort of hobbled in and actually walked out*, *I thought oh well perhaps it’s worth having my back checked…So ever since then I have been coming to see Ken*.*”*
**Ron, 30**	*“I live locally*. *Drove past this place a number of times and*, *speaking to Ken through the initial consultation I just thought it would be the best way to go*.*”*
**Joanne, 35**	*“I was having low back pain and it was getting increasingly worse…it was affecting my daily mobility*. *G*.*P*.*’s aren’t very good at stuff like that so I thought I’d seek an alternative treatment…*. *I passed daily on my way to and from work and I thought I would give him a try*.*”*

Several of the patients had clearly heard about chiropractic treatment through friends or relatives but there were also other mechanisms of referral. *Kate*, 36, was a patient of the resident herbalist and consulted the chiropractor after hurting her neck. *Liz*, also 36, had, after a serious car accident first seen an osteopath with limited success, and had then been recommended by a friend to consult Ken. For the majority of Ken’s patients recommendations from others appeared to have played a substantial part in the decision making process.

### Patients’ understanding of the mechanisms of treatment

Chiropractors spend considerable time on explanations and instructions with their patients. Kelner suggests that there are three types of explanations:

1. An explanation of the problem itself.

2. An explanation of treatment.

3. An explanation of chiropractic [35].

Discrepancies in the understanding of common health terms between clinicians and patients are often the reasons for poor communication [67], and patients’ beliefs and perceptions of treatment are factors that have been shown to influence treatment outcome [68,69]. Following on from this it has been suggested that patient-centred communication that facilitates the patient’s participation in decision making in relation to treatment has health promoting effects [46,56,70].

Since the nineteen seventies the traditional chiropractic concepts of *vertebral misalignment* have been challenged. One of the more contemporary explanations for musculo skeletal pain syndromes replacing them is based around joint mobility. While observing Ken with his patients I witnessed him repeatedly explaining that a lack of joint mobility was the cause of symptoms and that treatment would involve restoring normal mobility to the joints. I wanted to explore patients’ understanding of what was ‘wrong’ and how they interpreted the corrective mechanisms of chiropractic treatment (see Table 3).

**Table 3:** Patients’ understanding of the mechanism of chiropractic treatment

**Name andAge**	**Reply to the question: “What is your understanding of what Ken does?”**
**Sid, 46**	*“****Bjorn:****“So*, *what is your understanding of what Ken the chiropractor does?*....*”*
	***Sid:****“Well*, *basically to put it right in alignment…*.*You actually put the skeleton back to where it should be…*..*”*
**Uma, 60**	*“I presume that he tries to manipulate things back to where they should be…”*
**Liz, 36**	*“I hear nice crunches*, *so I guess sort of things are clicking back in and…*.*I suppose the way I think is mainly my spine is put out a little bit and it’s kind of slightly off and…he’s moving my bones back into place again*.*”*
**Pat, 26**	*“Just getting the vertebrae back into line by clicking them*, *that is all I really know*.*”*
**Ron, 30**	*“He said the lower muscles are quite tensed up*, *quite sort of fused…I need to do some exercises*, *where I go away and…stretch those muscles out and…*.*I think the vertebras that need to be sort of clicked back in place…and he sort of did three clicks today on the lower back on the left hand side and he just stretched me out…”*
**Mary, 50**	(Mary was receiving supportive treatment for stiffness and pain in her right hip joint as well as treatment of the spine. Her understanding involved the *mobility* of the joint). *“It’s releasing the things that are very*, *very tight…*.*He does the gentle massage bit first but…*.*the quick movements are like a release*. *That’s how it feels inside me; bits that have stuck together have been opened up a bit…*..*I am under the impression that the spine is just sort of compacted somewhat and whatever he does sort of releases that*.*”*
**Joanne, 35**	(Joanne used the term *unlocking* to describe the treatment of her lower back and pelvis)
	*“…Obviously the manipulation of the joints…*..*helps in movement in things…*.*that don’t work properly*.....*…*..*My pelvis was kind of locked and Ken did this manoeuvre and unlocks my pelvis so to speak*.*”*
**Dora, 51**	*“I have kind of read up on things…… not that well*. *I’m no expert…*.*and looked at alternative things as well and…it does make sense with the skeleton and how it works*, *and alignment………Ken stretching me and clicking me and that would keep you right as opposed to if you have an injury…*..*you find you work yourself the wrong way*.*”*
**Clare, 27**	*“From what I gather*, *if the joint*, *the muscles*, *the actual joints…don’t they affect the muscles which can cause the pain?……*.*I have never really asked him as to why or what the clicking of joints does*.*”*
**Tanya, 54**	*“He has said a bit about muscle and bones and shown me things…*.*He also uses terminology that makes me laugh because I don’t understand him…*.*”*
**Owen, 36**	(Owen, a trainee male nurse, correlated the effects of treatment to circulation). *“The joint manipulation from what I understand is…*.*opening a joint up a little bit…flowing blood supply in there*, *crushing it all up sort of thing*.*”*
**Vera, 46**	(Vera had little interest in understanding the mechanisms of treatment). *“The mechanics of it*, *I don’t understand*, *no*. *I leave that to Ken and I am assured by what and how he is that he is doing everything correctly*.*”*

Although some patients were clear that there were ‘right’ places and ‘wrong’ places for bones to ‘be’ the majority appeared to have a more fragmented understanding of the mechanisms of treatment. Vera’s answer illustrates the point that as a patient she was content to leave clinical decisions in the hands of her chiropractor. The effects and importance of patient centred care, trust, informed consent, and practitioner confidence on treatment outcome has been investigated and discussed in a number of studies [61,71,72]. It has also been suggested that the technical and interpersonal skills of the chiropractor in combination with the interaction, “the personality gel”, in the patient-clinician encounter in each consultation are factors that influence treatment outcome [33]. I asked Ken about his views on the traditional chiropractic concept of *spinal misalignment*:

*Ken: “That’s probably the ancient idea too isn’t it? Somebody would come in antalgic and* (*you*) *say ‘Your spine is crooked*, *I have just the thing for that’ and you apply some procedure*, *you hear it click and you can well imagine it is your spine clicking back into place*. *People still believe it now*, *don’t they?*......*It’s a lovely metaphor and you can see why it persists*.*”*

In spite of Ken’s considerable efforts in explaining what was at fault and how it would be corrected few patients appeared to have taken the information on board. For the majority of Ken’s patients the focus appeared to be on the positive outcome of treatment and many of them appeared surprised when I asked about their understanding of treatment mechanisms. Kelner writes: “Most patients adopt a pragmatic attitude toward chiropractic; they come to the practitioner because they have an ailment. For them the validity of chiropractic depends on the success that practitioners have in alleviating their miseries” [35]. *Nadine*, 28, illustrates this point in her succinct answer to my question on how the chiropractic treatment worked:

*“I don’t think I really think about it…As long as the pain goes*, *I don’t really mind what happens*.*”*

### Patients’ concerns

For most patients the first consultation with a chiropractor will involve elements that are unfamiliar and perhaps daunting. The majority of patients interviewed by me had been recommended to see Ken by someone in their immediate social environment and all patients that took part in interviews had undergone at least one treatment with him. During my observations at the Clinic it became increasingly clear that patients never doubted Ken’s good intentions. I asked Ken’s patients if they had been concerned or had experienced anxiety during any of their consultation and treatment visits. Although the majority of patients answered no to this question a small number had experienced some concerns (see Table 4).

**Table 4:** Patients’ concerns

**Name andAge**	**Reply to the question: “Were or are you concerned or anxious during consultations and treatment?”**
**Warren, 45**	*“****Warren:****…What I like about Ken as well is that he’s very…*.*What’s the word?…I’m a big guy and I am quite sort of cautious of who I take my shirt off in front of but my wife has come*, *and he is very sort of…*.*I can’t think of the word…Very professional and it’s a case of…*
	***Bjorn:****Respectful?*
	***W:****Absolutely*, *yeah*. *Here’s a gown for you and he’s out of the room*. *Not so much for me because I mean I just… get on with it…*
	***B:****Yes*.
	***W:****…But for like…He said to come in with the wife and so that she didn’t feel like*, *you know what I mean…*.*…So he was very respectful in that way*.*”*
**Ron, 30**	*“****Ron:****So…so yeah*, *there is*, *I think*, *for a lot of people there is the embarrassment factor as well*.
	***Bjorn:****Was that OK with you?*
	***R:****Yeah*, *it was OK with me…I think sometimes if you are dealing with another male it puts you at ease a little bit…*.*I think if you may be dealing with a female [chiropractor] then you may have in the back of your mind that you are a bit conscious about sort of taking your clothes off and things*.*”*
**Pat, 26**	(Pat was anxious about undressing and the amount of physical touch. In addition to this she was concerned about the manipulative treatment of the neck). *“Yeah*, *I was anxious…*.*just because I didn’t really know what needed to be done*, *you know*. *Just understanding it*, *or telling me to strip off…how much you need to touch me and things so …All that was completely new…but Ken reassured me and explained step by step what he was going to do and I was a bit concerned when I was lying down…He’s just gonna click my neck*. *But he gave it 2 or 3 minutes*, *you know*, *explaining what he was doing……*.*This is going to be loud because it’s close to your ear*, *and really telling exactly what was coming up*.*”*
**Vera, 46**	(Vera had reacted to the imposing appearance of the treatment table). *“I think*, *initially*, *that the beds were a bit…daunting…and you do worry how…well I don’t know*, *because I am quite confident and I am more relaxed with Ken*. *But you feel…*.*I felt quite embarrassed perhaps*, *like moving around…The gown thing and initially*. *I don’t have a problem with that at all now*.*”*

It would seem that once clinical routines had been set in motion most concerns that were initially present were diminished or disappeared entirely. It has been pointed out that physical treatment such as spinal manipulation requires the development of a substantial level of trust between practitioner and patient [73]. Successful treatment in the past is another factor linked to reduced levels of anxiety in patients [74]. In the section “The Healing Encounter” Kelner writes:

“Patients may evaluate treatment by other criteria as well as its effectiveness; for example, whether the experience is enjoyable or not. While one group of patients describes the experience as enjoyable, partly because of the physical contact (the laying on of the hands), and partly because of the euphoric after effects of the adjustments, there is another group who do not find it pleasant, being anxious about adjustments/manipulations……Chiropractic may also be judged in terms of patients’ beliefs. There are those who treat it as a mystical or religious experience, and there are those whose attitude is pragmatic, who go strictly for the measurable results. Lastly, there are those patients who believe in chiropractic for most health problems, while others remain dubious about its capacity to help any but a narrow range related to muscular/skeletal problems [35].”

The fact that patients are usually seen by the same chiropractor for the duration of treatment is another factor that reduces anxiety levels. It would seem that most patients prefer to stay with the same practitioner for the duration of treatment, or at least within the same clinic, (personal experience and communication with other chiropractors).

### The chiropractor’s approach

Early chiropractic concepts included the notion that the health of the spine directly affects the health of other organs and that chiropractic treatment could be applied to all forms of ill health and disease. There is still disagreement between different schools of thought within the chiropractic profession over the effectiveness of chiropractic treatment and the appropriate scope of practice of chiropractors. Different beliefs and opinions on these matters continue to fuel divisive debates within the profession. Although a unifying idea of chiropractic has been sought since the end of the nineteenth century it has been suggested that there may in fact not exist one concept that would encompass and accommodate the entire chiropractic profession [75]. During my observations of Ken he treated patients presenting with predominantly musculo-skeletal complaints. Treatment was carried out using spinal manipulative therapy, adjunctive therapies, and in addition to this advice to patients on activity, stretches and core-stability exercises.

Although treatment outcome does ultimately depend on the therapeutic intervention it is affected by a number of other factors surrounding the treatment visit [76]. It has been suggested that the following eight elements have a therapeutic effect and that they often form part of every clinical encounter:

1. Positive verbal reinforcement

2. Encouragement

3. Developing of Trust

4. Reassurance

5. Development of a Practitioner-Patient Relationship

6. Respect of Uniqueness

7. Exploration of Values

8. Creation of ceremony [77]

During my time as an observer in the Clinic I was witness to positive verbal reinforcement, encouragement, developing of trust, reassurance, and saw evidence of the relationship between Ken and his patients. In relation to elements 6 and 7 it was evident during my observations and in my conversations with Ken that he respected each of his patient’s individuality and “uniqueness”, and that he did search for “common values” during interactions with his patients. “Ceremony” was very much constantly a result of the greeting, questioning, examination, and treatment routines that formed the framework of every consultation. This could even be extended to include the activities in reception before and after each consultation. Kelner writes:

“While theoretical knowledge received a great deal of attention at the college, after chiropractors have been in practice for a while, it tends to decline in importance. It is treatment therapies and the accompanying social skills that take precedence with most practicing chiropractors……While debates about the philosophy of chiropractic and the validity of its theories of illness are of strategic importance in the political arena, they prove to be of little significance in the practice [35].”

As an experienced clinician Ken was aware of the wider impact of the clinical encounter and the difficulties in precisely defining the underlying mechanisms of the health benefits that occur in relation to chiropractic treatment. In one of our conversations we discussed the nature of the therapeutic mechanisms at play in chiropractic clinical practice:

*Ken: “We talk about getting people’s bodies right and getting to people’s ‘core’ to put it right and render motion right but…*.*You transfer*, *effect a change in that person’s wellbeing*, *and you try to communicate a change in that person’s wellbeing and the name of that change that we practice is ‘chiropractic’*.*”*

In his answer Ken had, in my opinion, made an attempt to define his own understanding of his role as a chiropractor and the *vision* or the *idea* underpinning his approach to chiropractic treatment and his clinical modus operandi. This fundamental idea of what chiropractic is and how it affects patients was probably unique to Ken. I suspect that each individual chiropractor would harbour his or her equally unique idea providing the basis for his/her understanding of and approach to chiropractic clinical practice.

## Conclusions

This Field Study, based on participant observation and semi-structured interviews, has provided an insight into the nature and characteristics of the clinical practice of a UK trained chiropractor at work in a suburban chiropractic clinic in western Great Britain. Despite its limitations there is, in the author’s opinion, valuable information to be extracted from this study.

The account describes the reception area suggesting its function in providing an effective and at the same time comforting prelude to the chiropractic consultation in this particular clinic. This field study has also described the initial chiropractic consultation and how the consultation room provides a private and secluded space suited to examination and treatment.

Earlier studies of chiropractic clinical practice have described the characteristics and elements that constitute the treatment visit. In this study the author was able to observe and describe the pre-treatment interview, examination, treatment, and re-assessment that form the basis of consultations at this particular clinic. This account also makes an attempt to describe the richness and nature of communication between the chiropractor and his patients in the consultation room. The author has attempted to illustrate how physical contact, both of an investigating and of a therapeutic nature, formed a substantial part of each chiropractic clinical encounter.

The patient interviews carried out by the author suggest that the majority of this chiropractor’s patients presented with pain of musculo-skeletal origin and that this, in combination with recommendations from relatives, friends, and work colleagues, constituted the principal reasons for these patients when making a decision to consult the chiropractor.

The majority of interviewed patients were found to have either an inaccurate or a rudimentary understanding of the mechanisms of chiropractic treatment and some patients even made it clear that they had little interest in treatment mechanisms. Despite the efforts made by this chiropractor in explaining why a patient would experience pain and how treatment worked most of these patients showed only a limited understanding of treatment mechanisms. This difference in patient attitudes and understanding of treatment has been identified by authors in earlier studies.

Only a small number of patients in this cohort reported concerns in relation to the treatment. This study suggests that any heightened anxiety levels were reduced as a result of the actions of the chiropractor and as individuals got accustomed to clinic routines.

In the interviews and conversations with the chiropractor the author was able to gain some insight into this chiropractor’s experiences, beliefs and opinions and how these provided the basis for his understanding of chiropractic treatment and his approach to chiropractic clinical practice.

This small-scale field study, despite being based on observations of a single chiropractor and a small number of his patients on one afternoon per week in one particular clinic, does suggest that the clinical chiropractic practice carried out by this particular UK trained British chiropractor contains a number of elements described in earlier qualitative studies of chiropractic clinical practice in the U.S., Canada, and Australia. Due to its size and the fact that the author is the sole observer, sole interpreter of observations and sole editor of interview transcripts, no robust conclusions can be drawn from this study. Further field work, both in depth and on a larger scale is, in the author’s opinion, necessary in order to establish whether or not the findings of this field study are more broadly represented in chiropractic clinical practice in the United Kingdom.

## Competing interests

The author declares that he has no competing interests.

## Supplementary Material

Additional file 1 Research Information Sheet. Click here for file

Additional file 2 Patient interviews. Click here for file

Additional file 3 Clinic Layout. Click here for file
