# Supplementary material for: Contemporary chiropractic practice in the UK: a field study of a chiropractor and his patients in a suburban chiropractic clinic
Source: Chiropr Man Therap. 2013 Aug 8;21:25. doi: 10.1186/2045-709X-21-25 (PMC3750721; doi:10.1186/2045-709X-21-25)
Supplement: Additional file 1 — Research Information Sheet. [file 2045-709X-21-25-S1.docx]

APPENDIX I

Research Information Sheet

________________________________________________________________

Clinic Research

I, BJORN HENNIUS, AM CONDUCTING A SERIES OF INTERVIEWS WITH PATIENTS UNDERGOING CHIROPRACTIC TREATMENT.

THE INTERVIEW SHOULD TAKE BETWEEN 15-20 MINUTES. QUESTIONS WILL REVOLVE AROUND THE REASONS FOR CHOOSING CHIROPRACTIC TREATMENT, THE TREATMENT ITSELF AND HOW TREATMENT HELPS.

THE INTERVIEW WILL BE RECORDED AND THE INFORMATION WILL BE USED FOR MY DISSERTATION AT BRUNEL UNIVERSITY.

YOUR PRIVACY AND CONFIDENTIALITY WILL BE FULLY RESPECTED. NO PERSONAL DETAILS OF ANY PATIENT INTERVIEWED WILL BE DISCLOSED IN THE TEXT ALTHOUGH THE DISSERTATION MAY GO TO PUBLICATION.

WHETHER YOU DECIDE TO TAKE PART OR NOT WILL IN NO WAY AFFECT YOUR TREATMENT.

Bjorn Hennius, Chiropractor
