# Supplementary material for: Contemporary chiropractic practice in the UK: a field study of a chiropractor and his patients in a suburban chiropractic clinic
Source: Chiropr Man Therap. 2013 Aug 8;21:25. doi: 10.1186/2045-709X-21-25 (PMC3750721; doi:10.1186/2045-709X-21-25)
Supplement: Additional file 2 — Patient interviews. [file 2045-709X-21-25-S2.docx]

**APPENDIX II**

**PATIENT INTERVIEWS**

- 1. HOW DID YOU END UP CONSULTING [KEN] THE CHIROPRACTOR?
  2. WHAT IS YOUR UNDERSTANDING OF WHAT [KEN] DOES?
  3. WERE OR ARE YOU CONCERNED OR ANXIOUS DURING CONSULTATIONS AND TREATMENT?
