# Supplementary figures and images for: Contemporary chiropractic practice in the UK: a field study of a chiropractor and his patients in a suburban chiropractic clinic
Source: Chiropr Man Therap. 2013 Aug 8;21:25. doi: 10.1186/2045-709X-21-25 (PMC3750721; doi:10.1186/2045-709X-21-25)

APPENDIX III

Clinic Layout


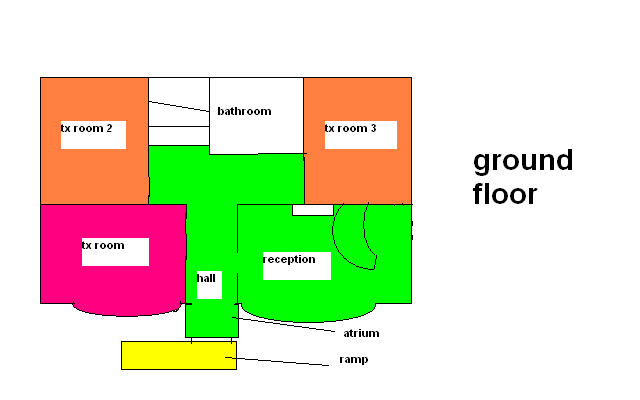


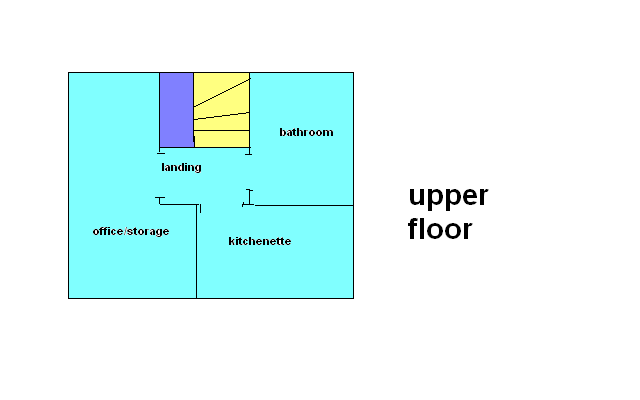

Supplement: Additional file 3 — Clinic Layout. [file 2045-709X-21-25-S3.docx]
